# The efficacy of genetic counselling for familial colorectal cancer. A randomised clinical trial

**DOI:** 10.1038/s41431-025-01921-x

**Published:** 2025-11-07

**Authors:** Andrada Ciucă, Tara Clancy, Sebastian Pintea, Ramona Moldovan

**Affiliations:** 1 Department of Psychology, Babeş-Bolyai University, Cluj-Napoca, Romania; 2 Division of Evolution and Genomic Sciences, School of Biological Science, University of Manchester, Manchester, UK; 3 Manchester Centre for Genomic Medicine, St Mary’s Hospital, Manchester University Hospitals NHS Foundation Trust, Manchester Academic Health Science Centre, Manchester, UK

**Keywords:** Colorectal cancer, Genetic counselling, Randomized controlled trials

## Abstract

Genetic counselling (GC) for familial colorectal cancer (fCRC) has been previously shown to improve outcomes such as emotional distress and screening adherence. This is the first randomised clinical trial to evaluate the efficacy of GC for fCRC. We included individuals affected or at-risk for fCRC (Lynch syndrome, APC-associated polyposis, other risk-associated pathogenic variants and clinically defined fCRC). Participants were randomised to (1) standard care or (2) standard care and genetic counselling. Measures include empowerment, anxiety, depression, knowledge, emotional distress, perceived social support, risk perception. Eighty-two individuals participated in the study. The average age was 44.81 years old, with 52.4% women. There was a significant effect in the counselling group (42/82) on post-intervention empowerment scores compared to the control group (40/82) (*p *= 0.004, *d *= 0.71), and similarly for depression (*p *= 0.025, *d *= 0.40), anxiety (*p *= 0.036, *d *= 0.35) and knowledge (*p *= 0.016, *d *= 0.25). Exploratory analysis show that several sociodemographic, affective and cognitive variables are moderating the improvement in empowerment following genetic counselling. Our data show significant improvements for both primary endpoint (empowerment) and secondary endpoints (knowledge, depression, anxiety, emotional distress). Genetic counselling is an effective intervention for fCRC both when the diagnosis is part of a syndrome or clinically defined, and both for affected or at-risk individuals.

## Introduction

Genetic counselling enables individuals to understand and adapt to the medical, psychological and familial implications of the genetic contribution to their condition [1]. Research shows that genetic counselling is an effective intervention for familial cancer [2] and it has a positive impact on knowledge [3], risk perception accuracy [3–5], anxiety, cancer-related worry, and decisional conflict [5].

Approximately 5% of CRC cases have a clear underlying genetic component [6]. Dominantly inherited conditions include Lynch syndrome (LS) which is associated with a 20–75% lifetime risk for developing CRC [7], familial adenomatous polyposis (FAP) with up to a 100% lifetime risk and the attenuated form of FAP (aFAP) with an ~70% lifetime risk [8, 9]. Familial CRC accounts for ~20–30% of all cases of CRC [6, 10] and is associated with up to a 3–6-fold CRC risk compared to the general population [11].

Existing research investigating the impact of genetic counselling for inherited and familial CRC shows promising results in terms of psychosocial outcomes [5, 12]. Psychological distress and anxiety in individuals after undergoing predictive testing for LS tend to increase after a positive result but return to the pre-test level at 6–12 months after test results disclosure [13]. Depression is a less studied outcome, but evidence suggests that it does not increase for unaffected individuals testing negative or returns to pre-test levels within 6 months of test result disclosure for unaffected individuals testing positive [13]. Individuals diagnosed with LS show an increase in psychological distress after the positive test disclosure but return to levels comparable with at risk unaffected individuals within 6 months of the disclosure [13]. Individuals who benefit from genetic counselling and test negative after predictive testing for LS appear to understand their risk to develop CRC more accurately than individuals who test positive [14, 15] and this difference is maintained up to 7 years post-test [16].

Genetic counselling has been shown to improve patient outcomes although few studies have investigated its effectiveness. The present trial is aimed to assess the efficacy of genetic counselling for inherited and familial CRC and to explore potential predictors of change that can have an impact on efficacy. The primary outcome of the trial is empowerment and secondary outcomes include anxiety, depression, emotional distress and knowledge. Outcomes were chosen based on the most frequent outcomes and measures used in cancer genetic counselling research to allow future comparisons between trials. Our aim is to enhance, through rigorous methodology, the evidence base for genetic counselling as an effective intervention and to address relevant outcomes for patients and their families.

## Methods

The trial was designed as a parallel 2-arm randomised controlled trial with 1:1 allocation ratio. The control arm consisted of usual care, which did not include genetic counselling and the experimental arm consisted of genetic counselling added to the usual care. The trial included recommendations from other randomised clinical trials in genetic counselling [12], and frequently used measures in genetic counselling research [17].

### Participants

The trial aimed to include (1) individuals (at risk or diagnosed) with a CRC with clear germline aetiology and (2) individuals (at risk or diagnosed) with familial CRC. We used the following inclusion criteria: (1) individuals from families with a confirmed inherited CRC syndrome; and in order to capture Lynch-like families we included (2) individuals from families with a first-degree relative diagnosed with CRC or endometrial cancer before age 50 (3) individuals from families with two or more first degree or second-degree relatives diagnosed with CRC or endometrial cancer regardless of age.

### Procedure

Participants were recruited between July 2019 and December 2022. The trial was reviewed and approved by the Institutional Review Board at the Oncology Institute in Cluj-Napoca (123/15.02.2019). The trial protocol was presented to clinical oncologists with details about eligibility criteria and information on referring patients to participate in the study; they received a printed poster and a short invitation letter to distribute it to potential participants. The trial was also advertised on social media and through printed posters. Once the patients were referred in consecutive order by oncologists, the first author (AC) arranged a first meeting to discuss the details of the trial. If they consented to participate, they were allocated to a group, and were asked to fill in the baseline questionnaires. G*power was used to calculate the sample size necessary to observe a difference between the experimental and control group. This analysis showed a total sample size of 60 participants divided into 2 equal groups of 30 participants in each would be needed. Participants were randomly allocated to one of the arms of the trial. The randomization sequence was generated with Randomization.com.

Affected individuals were recruited during their active cancer treatment period. For convenience, those allocated to the experimental group had their genetic counselling session scheduled in the same day as their next treatment appointment, usually 2–3 weeks after completing the baseline questionnaires. The post intervention questionnaires were given at the end of the session; participants were asked to complete and return these at the next treatment appointment, usually another 2–3 weeks later. For equivalence with the genetic counselling group, the control group completed the questionnaire at baseline and after two treatment sessions, usually 4–6 weeks after baseline. Participants were also informed which members of their family were also eligible to participate in the study. Participants could complete the entire set of questionnaires in a paper-and-pencil format or the equivalent online form.

### Intervention

Participants in the control arm received the usual care from their clinical oncologist, which did not include genetic counselling; genetic testing is usually discussed and can be arranged by oncologist; for complex or rare diagnoses, patients are referred to a clinical geneticist. None of the patients in the control group were seen by a geneticist. Participants in the experimental arm received the usual care and a genetic counselling session. The session took place face-to-face, lasted 60–80 min and was conducted by an EBMG board-certified genetic counsellor (AC). The content of the genetic counselling session is presented in Table 1.

**Table 1:** Text box with the content of the genetic counselling session.

• a brief introduction about genetic counselling
• a detailed recording of family history and relevant medical history
• information about cancer, the genetics of cancer, the process of genetic testing and possible results
• discussion about risks and implications for the individual and the family
• discussion about psychosocial issues and emotional concerns, information about the screening/management plan
• a plan for follow up

### Outcomes

#### Primary outcome

The primary outcome of the trial was empowerment, measured with the Genetic Counselling Outcome Scale (GCOS); this is a validated 24-item questionnaire, widely used in genetic counselling research [18]. In genetic settings, empowerment is defined as individuals’ beliefs that they can (1) make informed choices, (2) have sufficient information regarding the condition, risk, treatment and support, (3) use the health system for the benefit of the family, (4) manage the feelings associated with having a genetic condition, and (5) look at the future having hope [18]. GCOS scale was used for both groups with no adaptation made for the control group, to allow reliable comparisons between groups. The scoring is on a 7-point Likert scale that ranges from 1 = total disagreement to 7 = total agreement, with a middle option of 4 neither agreement nor disagreement. Given the rating system, no adaptation was necessary as the questions could be appropriately answered regardless of the group.

#### Secondary outcome

Several secondary outcomes were included to support the primary outcome and provide additional information. *Anxiety and Depression* were measured with the Hospital Anxiety and Depression Scale (HADS) [19]. HADS is a 14-item measure designed to evaluate anxiety and depression and is widely used in genetic counselling and interventional psychotherapy trials. *Emotional distress* was measured with the Impact of Event Scale (IES) [20]. IES is extensively used in genetic counselling as the risk for a disease is often conceptualized as a major stressor. IES is a 22-item scale designed to assess the level of intrusive and avoidant thoughts and hyperarousal related to a traumatic stressor.

As no validated scales were identified to measure knowledge and risk perception related to inherited and familial colorectal cancer, purposeful designed items were used. The items were created as a visual-analogue scale consisting of a line of 10 centimetres with 0 marked on the left side and 100 marked on the right side. *Knowledge* was self-reported with items that aimed to measure the level of knowledge about CRC and genetics, trust in the knowledge, and the utility of the knowledge. Participants were asked to what extent they considered they have enough knowledge about cancer, the level of trust in the knowledge they have, and if the knowledge they have is useful. *Risk perception* was measured to assess the perceived risk to carry a pathogenic variant associated with CRC, again using a visual-analogue scale.

*Perceived social support* was measured with the Multidimensional Scale of Perceived Social Support (MSPSS) [21]. The scale is designed to evaluate the sources of social support: family, friends or significant other.

Additionally, the participants completed a short sociodemographic questionnaire that included information about date of birth, family, occupation, education status, and diagnosis.

### Statistical analysis

#### Descriptive statistics of measured outcomes

Outcomes scores (means and standard deviations) were computed for genetic counselling and control groups for baseline and post-intervention.

#### Primary and secondary endpoints analysis

We tested for differences between groups at baseline and no significant differences for any of the outcomes were identified. We conducted *paired sample t-tests* to investigate differences between baseline and post-interventions for the control and counselling group. For this, we performed a Bonferroni correction for multiple comparisons; the adjusted *p*-value threshold for this analysis is 0.0055. A negative t-test coefficient usually implies that the scores at pre-interventions are lower than the scores at post-intervention. We also conducted a *covariance analysis* to control the observed differences for the baseline scores; this analysis is designed to remove the differences in post-intervention scores which can be attributed to differences in the baseline to increase the accuracy of comparisons and increase the validity of the study leading to a more robust estimation of the counselling effect. The values of Cohen’s d can be interpreted as follows: 0.2 suggests a small effect size, 0.5 constitutes a medium effect size, and 0.8 is large effect size.

## Results

**Fig. 1:**
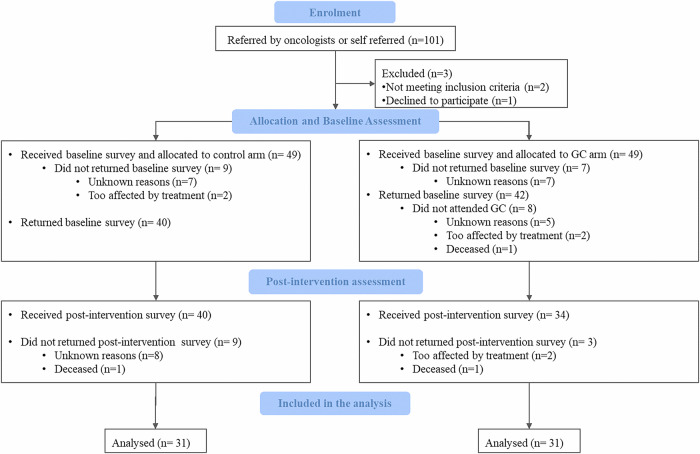
Flow of participants enrolment and participation.

Overall, 101 individuals were referred by oncologists or self-referred to participate in the trial. Of these, 2 individuals did not meet the inclusion criteria and 1 declined to participate. After randomisation, 49 participants were allocated to the genetic counselling group and 49 to the control group and all received the baseline questionnaires. In the counselling group, 42 individuals returned the baseline questionnaire and 34 attended the genetic counselling session. In the control group, 40 individuals returned the baseline questionnaire. The post-intervention questionnaire was returned by 31 participants in the counselling group and 31 in the control group. Figure 1 presents the flow of participant enrolment and participation through the study. In total, 82 participants completed the baseline questionnaire and 62 the post-intervention questionnaire.

### Participants characteristics

The baseline questionnaire was filled in by 82 participants. The average age of the participants was 44.81 years (SD = 11.40). In total, 39 men and 43 women participated in the trial. Most participants (*n *= 56) had familial CRC, some (*n* = 18) had a confirmed or suspected genetic diagnosis (Lynch Syndrome, FAP/aFAP, Li-Fraumeni/TP53, MAP/MUTYH, PALB2) and a few (*n* = 8) had other cancer diagnoses (endometrial, breast, pancreatic, thyroid) and a family history of CRC. The majority of the participants (*n *= 71) were affected by cancer and the trial overlapped with the treatment period. A small proportion (*n* = 11) of the participants were unaffected at-risk individuals. Table 2 presents the detailed sociodemographic characteristics of the individuals participating in the trial.

**Table 2:** Participants characteristics.

Characteristic	Genetic counselling group (*n* = 42)		Treatment as usual group (*n* = 40)		Comparisons	
	Mean	SD	Mean	SD	*t*	*p*
Age of participants	44.80	11.25	44.82	11.72	0.01	0.99
	%	*N*	%	*N*		
Gender					−1.31	0.19
Man	40.5	17	55.0	22		
Woman	59.5	25	45.0	18		
Diagnosis (affected & at risk)					0.64	0.52
Clinically defined fCRC	69.0	29	67.5	27		
CRC predisposing syndromes/genes (Lynch Syndrome, FAP/aFAP, TP53, MUTYH, PALB2)	19.1	8	25.0	10		
Other diagnoses with FH of CRC (Endometrial, breast, pancreatic, thyroid)	11.9	5	7.5	3		
Genetic status					−0.23	0.81
Affected	85.7	36	87.5	35		
At risk	14.3	6	12.5	5		
Relationship status					−1.14	0.25
In a relationship	73.8	31	85.0	34		
Divorced	9.5	4	5.0	2		
Single	16.7	7	10.0	4		
No. of children					−0.23	0.81
none	23.8	10	32.5	13		
one	33.3	14	30.0	12		
two	35.7	15	27.5	11		
three or more	7.2	3	10.0	4		
Education					−0.76	0.44
Middle school	4.8	2	2.5	1		
High school	33.3	14	47.5	19		
University degree	61.9	26	50.0	20		
Occupation					0.20	0.83
Student	2.4	1	5	2		
Unemployed	4.8	2	7.5	3		
Employed	71.4	30	55.0	22		
Retired	21.4	9	32.5	13		
Living area					3.20	0.002
Urban	85.7	36	55.0	22		
Rural	14.3	6	45.0	18		
Religion					−0.51	0.60
Orthodox Christian	73.8	31	77.5	31		
Not religious	4.8	2	7.5	3		
Other	21.4	9	15.0	6		

Descriptive statistics of measured outcomes are presented in Table 3.

**Table 3:** Means and standard deviations for all measures by group at pre- and post-intervention assessments.

Measure	Genetic Counselling Group				Treatment as Usual Group			
	Pre-intervention (*n* = 42)		Post-intervention (*n* = 31)		Pre-intervention (*n* = 40)		Post-intervention (*n* = 31)	
	Mean	SD	Mean	SD	Mean	SD	Mean	SD
GCOS	117.47	17.11	127.64	14.86	114.90	15.83	115.19	19.48
Anxiety (HADS)	6.73	4.08	5.64	3.75	7.70	3.18	6.90	3.30
Depression (HADS)	5.11	3.35	4.12	2.75	5.92	3.64	5.54	4.14
Impact of event scale	29.57	14.04	26.22	15.70	31.65	14.23	27.90	14.42
Knowledge level	43.33	23.26	54.67	22.08	42.75	25.54	48.55	26.11
Knowledge trust	49.29	26.65	63.00	24.55	47.63	27.05	51.94	30.59
Knowledge utility	53.21	27.91	65.00	23.48	54.63	28.98	59.35	29.29
Risk perception	41.32	32.58	40.70	28.13	34.48	35.99	39.19	33.51
Perceived social support	78.59	8.42	77.90	5.76	76.90	10.65	76.67	9.93

Primary and secondary endpoints analysis and coefficients are presented in Table 4.

**Table 4:** Statistical analysis coefficients.

Statistical test	Paired sample *t*-test (pre vs post)						ANCOVA		
	Control group			Counselling group			Baseline scores as covariates		
	*t*	*p* (0.0055)	*d*	*t*	*p* (0.0055)	*d*	*F*	*P* (0.05)	*d*
GCOS	−0.028	0.978	0.005	−4.069	**0.000**	0.731	8.783	**0.004**	0.711
Anxiety - HADS	1.699	0.100	0.305	3.124	**0.004**	0.561	4.623	**0.036**	0.353
Depression - HADS	0.000	1.000	0.000	3.268	**0.003**	0.587	5.325	**0.025**	0.406
Impact of event scale	2.061	0.048	0.370	2.247	0.032	0.410	0.108	0.744	0.088
Knowledge level	−2.278	0.030	0.409	−3.961	**0.000**	0.723	6.182	**0.016**	0.250
Knowledge trust	−2.697	0.011	0.484	−2.631	0.013	0.480	1.146	0.289	0.387
Knowledge utility	−2.258	0.031	0.406	−2.622	0.014	0.479	0.186	0.668	0.208
Perceived social support	0.484	0.632	0.087	2.100	**0.044**	0.377	0.990	0.324	0.149
Risk perception	−1.236	0.226	0.222	0.427	0.673	0.078	0.830	0.366	0.048

The values in bold are above the established statistical significance threshold.

### Primary outcome

**Fig. 2:**
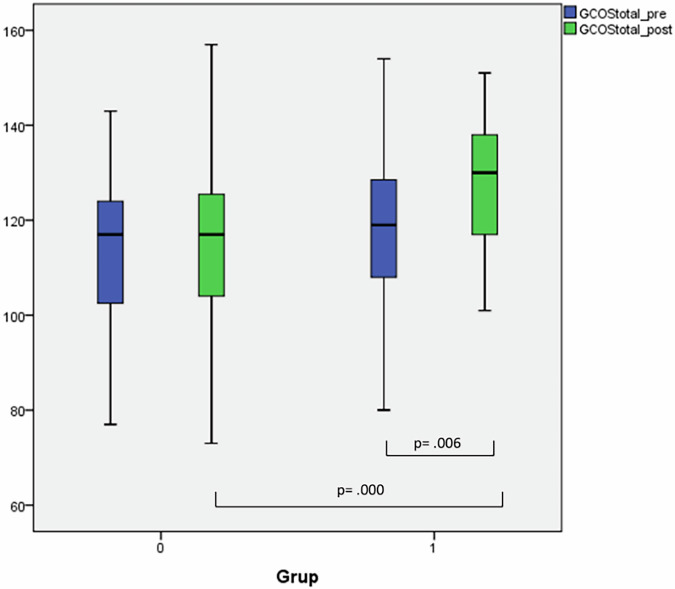
GCOS scores.

Our data show significantly higher empowerment when both comparing the scores after the intervention between the two groups and when comparing the scores before and after the intervention in the genetic counselling group. Figure 2 shows the empowerment scores at pre- and post-intervention for the two groups. A one-way ANCOVA was conducted to investigate the difference in post-intervention empowerment scores between groups when controlling for pre-intervention empowerment scores. There is a significant effect in the genetic counselling group on post-intervention empowerment scores after controlling for pre-intervention scores. The effect size is medium to large (*d *= 0.711). For detailed coefficients see Table 4.

#### Secondary outcomes

Anxiety and depression were improved after genetic counselling with an effect size of small to medium for anxiety (*d *= 0.353) and for depression (*d *= 0.406); emotional distress did not reach the statistically significant threshold. The perceived level of knowledge increased after genetic counselling, with a small effect size (*d *= 0.250). Perceived social support showed improvement in the genetic counselling group but did not reach statistically significant threshold after covariance analysis, similarly for risk perception. For detailed coefficients see Table 4.

### Exploratory analyses: predictors of change for empowerment

We used regression analysis to test the predictive values of several variables on the change in GCOS scores. The living area (urban/rural) was a predictor of change for GCOS (β = −7.32, t(60) = −2.26, *p *= 0.027) with participants living in urban areas having higher scores. Also, having/not having a diagnosis was a predictor of change; individuals with a diagnosis showing improved scores (β = 9.00, t(60) = 2.35, *p *= 0.022). Anxiety was a strong predictor of change for empowerment, and anxiety scores at pre (β = −0.80, t(60) = −1.94, *p *= 0.05) and post intervention (β = −1.380, t(60) = −3.42, *p *= 0.001) were predictors of the change in GCOS scores. Furthermore, the change in anxiety predicted a greater increase in empowerment (β = −2.091, t(60) = −2.50, *p *= 0.015). Emotional distress was also a predictor of change for empowerment; scores at both pre (β = −0.266, t(60) = −2.49, *p *= 0.016) and post intervention (β = −0.225, t(60) = −2.23, *p *= 0.029) predicted the change in GCOS scores. The trust that participants had in their knowledge after the intervention also predicted the change in empowerment (β = 0.117, t(60) = 2.14, *p *= 0.036); participants with greater trust in their knowledge after the intervention also had higher levels of empowerment. Participants with a high initial perception of their risk of carrying a pathogenic variant had greater improvements in empowerment (β = 0.115, t(60) = 2.50, *p *= 0.015). Finally, perceived social support at pre-intervention predicted the change in empowerment (β = −0.372, t(60) = −2.08, *p *= 0.041).

## Discussion

The aim of this trial was to investigate the efficacy of genetic counselling for inherited and familial CRC. To achieve this, we designed a randomised controlled trial to compare a genetic counselling session added to the treatment as usual with treatment as usual alone. We assessed the efficacy of the session by measuring empowerment with the GCOS scale, the most frequently used measure in genetic counselling research. Additionally, we looked at the impact of genetic counselling on anxiety, depression, knowledge, emotional distress, perceived social support, and risk perception.

### Primary endpoint

Data analysis shows genetic counselling was effective in improving empowerment for individuals at risk or affected by inherited and familial CRC. Looking only at the genetic counselling group, we observed a statistically significant increase in empowerment compared with the baseline scores. When comparing empowerment between both groups, we observed a statistically significant improvement in the counselling group following the genetic counselling session. This is in line with other prospective studies looking at empowerment in cancer clinics [22, 23]. The setting of the study provided a unique opportunity to investigate genetic counselling added to a treatment as usual condition, which does not regularly include genetic counselling. In order to evidence genetic counselling efficacy, we analysed the data by comparing the after-intervention levels of empowerment by controlling for the baseline levels. This gold standard analysis in RCTs allows us to remove potential baseline differences from the observed post-intervention differences. The significant differences were maintained after controlling for the baseline scores. This provides robust evidence to support genetic counselling as an effective intervention to improve empowerment in individuals at risk of or affected by inherited and familial CRC. Results from previous studies indicate this as an expected result, with genetic counselling consistently improving psychosocial outcomes in inherited and familial CRC setting [16, 24, 25].

### Secondary endpoints

A single measure cannot encompass all possible genetic counselling impacts, therefore we looked at several other relevant outcomes to more comprehensively capture the genetic counselling effect.

Improvement in depression was an expected result and it is in line with the majority of previous research investigating depression as a genetic counselling outcome. Previous data shows a significant decrease or a decreasing trend in depression after genetic counselling and testing regardless of genetic test result [13, 24–28]. All previous studies investigated depression in a genetic testing context and measured it before and after genetic counselling and testing. The novelty brought by the present trial is that it investigates depression in relation to genetic counselling alone and compares the results with a control group.

Improvement in anxiety was also in line with previous research showing that in a genetic testing context anxiety decreases after a negative test result and short increases in anxiety are followed by decreases after a positive test result with a return to baseline after 4–6 months [13, 16, 24, 25, 27]. Studies investigating anxiety in genetic counselling settings without genetic testing showed that gender and age have an impact on anxiety, with a significant reduction in anxiety for women regardless of age and men above 50 years but no improvement in younger men [29] and a less prominent decrease in anxiety in affected individuals compared with unaffected at-risk individuals [30]. Taken together these results highlight the complex interactions influencing anxiety in relation to genetic counselling and testing for CRC.

Emotional distress in previous studies using the same measure for emotional distress was found to decrease or increase depending on genetic test result following genetic counselling and testing [25, 27]. Improvement in emotional distress after genetic counselling might be influenced by diagnosis status and gender, with women having higher distress than men; previous data have also shown that affected individuals had significantly higher distress than unaffected individuals after genetic counselling and testing, associated with receiving a cancer diagnosis [24]. Complex interactions of genetic and diagnosis status or gender might influence improvements in emotional distress during the genetic counselling process.

Improvement in knowledge was also a result we expected, as previous research focusing on the efficacy of genetic counselling for CRC support similar findings [30–32].

### Effect sizes

Our data show that the magnitude of change brought by genetic counselling for CRC is similar with other meta-analytical reports. For anxiety, a similar effect size (r = −0.17) is reported after genetic counselling for breast cancer [2] whilst other reports showing no effect on anxiety (pooled long-term effect = 0.05 U) [3]. Similarly, our trial showed an increased perceived level of knowledge, and this is similar to a meta-analysis on familial cancer where similar results were reported (pooled short-term difference = 0.70 U) [3]. In terms of risk perception, our trial showed no significant change following genetic counselling, and this is similar to other meta-analyses (pooled short-term difference = −0.10 U) [3], but contrasting with other reports showing improvements in risk perception accuracy r = 0.56 [2].

### Predictors of change in empowerment

To gain a more in-depth understanding of the genetic counselling effect we looked at variables that are predictors of change for empowerment. A sociodemographic variable that had an impact on empowerment was participants’ residence, with individuals living in urban areas showing a significantly higher increase in empowerment after genetic counselling. This effect might be explained by differences in health behaviours and access to health services: populations in urban areas are known to have higher health literacy [33] and easier access to cancer genetic services [34] compared to rural areas. Another variable that impacted the effect of genetic counselling on empowerment was the type of diagnosis, with a syndrome diagnosis (suspected or confirmed) being associated with a greater increase in empowerment; this may have also been impacted by the phrasing of some GCOS items which might be more tailored for individuals having a clear genetic diagnosis. Two affective variables, anxiety and emotional distress, predicted the change of empowerment after genetic counselling. Participants with higher anxiety and emotional distress before and after the intervention had a smaller increase in empowerment. The higher the trust participants had in their knowledge after genetic counselling and the higher the initial risk perception of having a positive test result predicted an increased level of empowerment after genetic counselling. An exploratory variable that predicted the change after genetic counselling was perceived social support, with participants with initial lower perceived social support had a higher increase in empowerment after genetic counselling. This might be due to the active encouragement during genetic counselling sessions to seek and maintain a strong support network (i.e., family or medical team) to better cope with the implications of the cancer diagnosis which in turn could increase the empowerment individuals feel they have. More research is needed to gain more insight into the means by which genetic counselling leads to better outcomes for patients.

### Limitations and future directions

Our trial has some limitations. The heterogeneity of participants is relatively increased because the participants had very diverse diagnoses of CRC in terms of stage, treatment, and time since diagnosis. Another limitation could be the timing of the genetic counselling session in relation to the cancer treatment as they overlapped for most of the participants. Both of these aspects may contribute to the changes in affective and cognitive outcomes outside of genetic counselling session.

The outcomes included in the trial were selected based on the most frequent outcomes and measures used in genetic counselling research to allow comparisons between trials. Future trials can consider improving the trial design to include more programmatically chosen outcomes based on innovative approaches such as core outcome sets (COS) [35].

## Conclusions

The findings of the present trial robustly support the efficacy of genetic counselling for inherited and familial CRC. The primary outcome, empowerment, improved after genetic counselling when compared to the control group and after controlling for baseline levels. Affective outcomes findings are in line with previous research showing significant improvement in depression and anxiety levels. Perceived knowledge showed significant improvements at post intervention. Exploratory analysis show that several sociodemographic, affective and cognitive variables are predictors of change and contribute to the improvement in empowerment following genetic counselling.

Our findings evidence that genetic counselling increases empowerment and leads to measurable psychological benefits. This trial highlights the benefits of genetic counselling as an evidence-based intervention tailored to enhance a wide range of relevant psychosocial outcomes for individuals affected or at risk for CRC.

## Data Availability

Data are available from the corresponding author on reasonable request.
